# Serum 25-Hydroxyvitamin D Concentrations and Major Depression: A Mendelian Randomization Study

**DOI:** 10.3390/nu10121987

**Published:** 2018-12-15

**Authors:** Karl Michaëlsson, Håkan Melhus, Susanna C. Larsson

**Affiliations:** 1 Department of Surgical Sciences, Uppsala University, SE-751 85 Uppsala, Sweden; 2 Department of Medical Sciences, Uppsala University, SE-751 85 Uppsala, Sweden; hakan.melhus@medsci.uu.se; 3 Unit of Cardiovascular and Nutritional Epidemiology, Institute of Environmental Medicine, Karolinska Institutet, SE-171 77 Stockholm, Sweden

**Keywords:** depression, Mendelian randomization, single nucleotide polymorphisms, vitamin D

## Abstract

Whether vitamin D insufficiency is a contributing cause of depression remains unclear. We assessed whether serum 25-hydroxyvitamin D (S-25OHD) concentrations, the clinical marker of vitamin D status, were associated with major depression using Mendelian randomization. We used summary statistics data for six single-nucleotide polymorphisms significantly associated with S-25OHD concentrations in the Study of Underlying Genetic Determinants of Vitamin D and Highly Related Traits (SUNLIGHT) consortium and the corresponding data for major depression (*n* = 59,851 cases and 113,154 controls) from the Psychiatric Genomics Consortium. Genetically predicted S-25OHD concentrations were not associated with major depression. The odds ratio per genetically predicted one standard deviation decrease in S-25OHD concentrations was 1.02 (95% confidence interval 0.97–1.08; *p* = 0.44). The results of this study indicate that genetically lowered S-25OHD concentrations are not associated with increased risk of developing major depression.

## 1. Introduction 

Major depressive disorders are one of five leading causes of morbidity, with a global annual estimated total of 34 million years lived with disability [1]. A suggested contributing cause for the development of major depression is vitamin D insufficiency, and vitamin D supplementation is promoted to reduce risk of depression [2,3]. Vitamin D receptors are widely distributed throughout the human brain, including limbic structures likely involved in the regulation of mood. Calcitriol, the active form of vitamin D, can influence synthesis of catecholamines and serotonin implicated in the development of mood disorders, and findings from animal studies suggest that vitamin D may alter mood behavior [2,3]. Epidemiologic studies indicate a linear inverse relationship of vitamin D status, determined by serum 25-hydroxyvitamin D (S-25OHD) concentrations, with depression, but a reverse causation phenomenon may explain the connection [2]. There have been few double-blind randomized clinical trials, and generally (with some exceptions) negative results have been observed, although the vitamin D intervention periods have lasted only a few years [2].

Mendelian randomization is an epidemiologic study design in which genetic variants with an impact on the modifiable exposure of interest (e.g., S-25OHD concentrations) are used as instrumental variables to determine whether the exposure causes the disease (e.g., depression). Compared with ordinary observational studies, Mendelian randomization studies are less likely biased by confounding and are not influenced by reverse causality. Confounding is mitigated in a Mendelian randomization study because genetic variants are randomly assorted at conception and therefore unlikely related to potential confounding variables. Moreover, disease outcome cannot modify genotype, which is fixed at conception, thereby avoiding reverse causation bias. 

The Mendelian randomization design has not yet been applied to investigate whether S-25OHD concentrations are associated with major depression. We thus conducted a two-sample Mendelian randomization study using genetic variants that influence S-25OHD concentrations to determine the association of life-long decreased S-25OHD concentrations with major depression. 

## 2. Methods

### 2.1. SNP Selection and Data Sources

We selected all six independent single-nucleotide polymorphisms (SNPs) previously found to be associated with S-25OHD concentrations at genome-wide significance (*p* < 5 × 10^−8^) in the Study of Underlying Genetic Determinants of Vitamin D and Highly Related Traits (SUNLIGHT) consortium, including 79,366 individuals of European ancestry [4]. Together, these SNPs explain 7.5% of the variance in S-25OHD concentrations. Summary statistics data for the association between the six SNPs and major depression were obtained from the Psychiatric Genomics Consortium (Major Depression 2 genome-wide association study), which included 59,851 cases with major depression and 113,154 controls (Table 1) of European ancestry (not including 23andMe) [5]. Cases were required to meet international consensus criteria (Diagnostic and Statistical Manual of Mental Disorders, Fourth Edition, International Classification of Diseases, Ninth and Tenth Revision). There was no overlap between studies included in the SUNLIGHT consortium and those in the Psychiatric Genomics Consortium.

### 2.2. Statistical Analysis

We calculated an instrumental variable ratio estimate for each SNP by dividing the beta-coefficient for the SNP–depression association by the beta-coefficient for the SNP–25OHD association. The ratio estimates were combined using the inverse-variance weighted method (standard Mendelian randomization method) [6]. As a sensitivity analysis, we used the weighted median method, which is less sensitive to outlying genetic variants. The MR-Egger regression method was used to assess pleiotropy [6], that is, when a genetic variant is related to more than one phenotype. The analyses were performed in Stata (StataCorp, College Station, TX, USA) using the mrrobust package. Reported odds ratios (ORs) are expressed per one standard deviation (SD) decrease of S-25OHD concentration. An approximate SD for S-25OHD was obtained from the Swedish Mammography Cohort Clinical study [7] and was 0.33 ln-nmol/L (about 18 nmol/L). All statistical tests were two-sided, and a *p* value below 0.05 was considered statistically significant. Statistical power was calculated using a web-based application [8]. The power calculation was based on sample size, proportion of cases of major depression, proportion of variance explained by the S-25OHD-associated SNPs, and a type-I error rate (*α*) of 0.05. Heterogeneity among SNPs was tested using the *I*^2^ statistic [9].

## 3. Results

**Figure 1:**
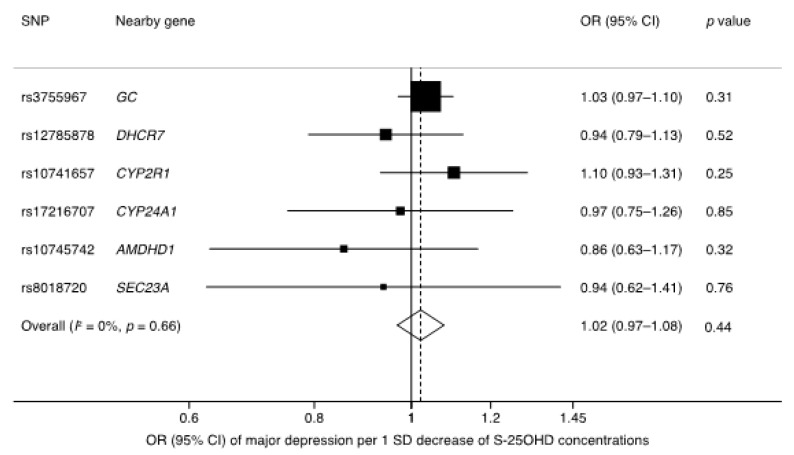
Association of genetically predicted one SD decrease of S-25OHD concentrations with major depression. S-25OHD, serum 25-hydroxyvitamin D; CI, confidence interval; OR, odds ratio; SD, standard deviation; SNP, single-nucleotide polymorphism.

We had 100% power to detect a 9% higher odds of major depression. Genetically higher S-25OHD concentration was not associated with major depression, either as single SNPs (Table 1) or combined as a summary measure (Figure 1). The OR per genetically predicted one SD (18 nmol/L) decrease of S-25OHD concentrations was 1.02 (95% confidence interval [CI] 0.97–1.08; *p* = 0.44), with no heterogeneity among individual SNPs (*I*^2^ = 0%) (Figure 1). Results were similar in sensitivity analyses based on the weighted median (OR 1.03; 95% CI 0.97–1.09; *p* = 0.39) and MR-Egger (OR 1.06; 0.97–1.17; *p* = 0.21) methods. Furthermore, there was no evidence of pleiotropy (MR-Egger intercept: −0.006; *p* = 0.33).

## 4. Discussion

Using six independent SNPs as instruments, we for the first time applied a two-sample Mendelian randomization approach to determine the causal nature of the association of S-25OHD concentrations with major depression. Our null findings indicate that there exists no association between lowered S-25OHD concentrations and increased risk of major depression, which contrasts results from ordinary observational studies [2,3]. Accordingly, with a stronger study design, we failed to find evidence that vitamin D status on average is related to risk of major depression.

Although our Mendelian randomization study permits causal inferences, it also has some potential limitations. Because our analysis was restricted to populations of European ancestry, we cannot make any assumptions concerning the association between S-25OHD concentrations and major depression in non-European populations. Moreover, we had limited power to examine effects of extremes on either side of the S-25OHD spectrum. Finally, as in any Mendelian randomization analysis, we cannot rule out the possibility that pleiotropy (i.e., when a genetic variant is associated with more than one phenotype) has affected the results and that the S-25OHD-associated variants influence major depression through a pathway other than S-25OHD concentrations. However, given that none of the variants were associated with major depression and that there was no indication of directional pleiotropy in the MR-Egger analysis, the results of this study provide no evidence to support this possibility. 

## 5. Conclusions

We conclude that life-long decreases in S-25OHD concentrations do not seem to increase the risk of major depression in generally healthy populations. Whether vitamin D supplementation may provide a benefit to those with very low vitamin D status is still uncertain [10]. 

## Figures and Tables

**Table 1:** Characteristics of the single-nucleotide polymorphisms (SNPs) associated with S-25OHD concentrations and their associations with major depression.

					Association with S-25OHD			Association with Major Depression				
SNP	Chr	Nearby Gene	EA *	EAF	β ^†^	SE	*p* Value	β ^‡^	SE	*p* Value	Cases	Controls
rs3755967	4	*GC*	T	0.28	−0.089	0.002	4.74 × 10^−343^	0.009	0.009	0.31	59,851	113,154
rs12785878	11	*DHCR7*	G	0.25	−0.036	0.002	3.80 × 10^−62^	−0.006	0.010	0.52	45,591	97,674
rs10741657	11	*CYP2R1*	G	0.60	−0.031	0.002	2.05 × 10^−46^	0.009	0.008	0.25	59,851	113,154
rs17216707	20	*CYP24A1*	C	0.21	−0.026	0.003	8.14 × 10^−23^	−0.002	0.010	0.85	59,851	113,154
rs10745742	12	*AMDHD1*	C	0.60	−0.017	0.002	1.88 × 10^−14^	−0.008	0.008	0.32	59,851	113,154
rs8018720	14	*SEC23A*	C	0.82	−0.017	0.003	4.72 × 10^−9^	−0.003	0.011	0.76	58,854	106,796

Chr, chromosome; EA, effect allele; EAF, effect allele frequency; S-25OHD, serum 25-hydroxyvitamin D; SE, standard error. * Allele associated with lower S-25OHD concentrations. ^†^ β coefficient of the S-25OHD-decreasing allele on natural log-transformed S-25OHD concentrations. ^‡^ β coefficient of the S-25OHD-decreasing allele on log odds ratio of major depression.
